# Supplementary material for: Resolving the full spectrum of human genome variation using Linked-Reads
Source: Genome Res. 2019 Apr;29(4):635–45. doi: 10.1101/gr.234443.118 (PMC6442396; doi:10.1101/gr.234443.118)
Supplement: Supplemental Material [file supp_gr.234443.118_Supplemental_longranger-2.2.zip › longranger-2.2.tar.bz2!/lr2.2-public/lib/go/src/code.google.com/vitess/vitess.io/_layouts/article.html]

---
layout: default
---

{% if page.image.feature %}

{% if page.image.credit %}{% include image-credit.html %}{% endif %}

{% endif %}
{% include breadcrumbs.html %}

# {{ page.title }}

{{ content }}

---


{% if page.categories %}{% include page-author.html %}{% endif %}
{% if page.share != false %}{% include share-this.html %}{% endif %}
{% include page-meta.html %}

{% if page.comments == true %}{% include comments.html %}{% endif %}
